# (*E*)-1-(4-Decyl­oxyphen­yl)-3-(4-hydroxy­phen­yl)prop-2-en-1-one

**DOI:** 10.1107/S160053680901054X

**Published:** 2009-03-28

**Authors:** Ibrahim Abdul Razak, Hoong-Kun Fun, Zainab Ngaini, Siti Muhaini Haris Fadzillah, Hasnain Hussain

**Affiliations:** a X-ray Crystallography Unit, School of Physics, Universiti Sains Malaysia, 11800 USM, Penang, Malaysia; b Department of Chemistry, Faculty of Resource Science and Technology, Universiti Malaysia Sarawak, 94300 Kota Samarahan, Sarawak, Malaysia; c Department of Molecular Biology, Faculty of Resource Science and Technology, Universiti Malaysia Sarawak, 94300 Kota Samarahan, Sarawak, Malaysia

## Abstract

In the title compound, C_25_H_32_O_3_, the asymmetric unit contains two crystallographically independent mol­ecules: both enone groups adopt an *s*-*cis* configuration. In the crystal, O—H⋯O and C—H⋯O inter­molecular inter­actions form bifurcated hydrogen bonds, which generate *R* ^1^_2_(6) ring motifs. These inter­molecular inter­actions link the mol­ecules into one-dimensional chains along the [10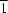] direction. The crystal structure is further stabilized by C—H⋯π inter­actions.

## Related literature

For general background to the biological properties of chalcone derivatives, see: Bhat *et al.* (2005 ▶); Xue *et al.* (2004 ▶); Satyanarayana *et al.* (2004 ▶); Zhao *et al.* (2005 ▶); Yayli *et al.* (2006 ▶). For related structures, see: Ng, Razak *et al.* (2006 ▶); Ng, Patil *et al.* (2006 ▶). For details of hydrogen-bond motifs, see: Bernstein *et al.* (1995 ▶). For bond-length data, see: Allen *et al.* (1987 ▶). For the stability of the temperature controller used in the data collection, see: Cosier & Glazer (1986 ▶).
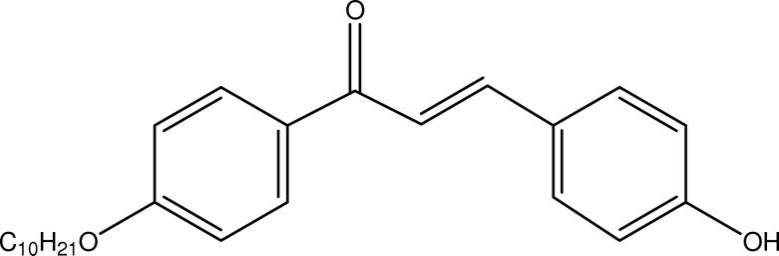


## Experimental

### 

#### Crystal data

C_25_H_32_O_3_
*M* *_r_* = 380.51
Monoclinic, 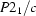

*a* = 12.4437 (2) Å
*b* = 35.5191 (6) Å
*c* = 9.8004 (2) Å
β = 99.284 (1)°
*V* = 4274.93 (13) Å^3^
*Z* = 8
Mo *K*α radiation
μ = 0.08 mm^−1^
*T* = 100 K
0.52 × 0.44 × 0.35 mm

#### Data collection

Bruker APEXII CCD area-detector diffractometer
Absorption correction: multi-scan (*SADABS*; Bruker, 2005 ▶) *T*_min_ = 0.962, *T*_max_ = 0.974
62626 measured reflections
16928 independent reflections
12634 reflections with *I* > 2σ(*I*)
*R*_int_ = 0.027

#### Refinement

*R*[*F* ^2^ > 2σ(*F* ^2^)] = 0.056
*wR*(*F* ^2^) = 0.153
*S* = 1.04
16928 reflections
515 parameters
H atoms treated by a mixture of independent and constrained refinement
Δρ_max_ = 0.54 e Å^−3^
Δρ_min_ = −0.25 e Å^−3^

### 

Data collection: *APEX2* (Bruker, 2005 ▶); cell refinement: *SAINT* (Bruker, 2005 ▶); data reduction: *SAINT*; program(s) used to solve structure: *SHELXTL* (Sheldrick, 2008 ▶); program(s) used to refine structure: *SHELXTL*; molecular graphics: *SHELXTL*; software used to prepare material for publication: *SHELXTL* and *PLATON* (Spek, 2009 ▶).

## Supplementary Material

Crystal structure: contains datablocks global, I. DOI: 10.1107/S160053680901054X/at2746sup1.cif

Structure factors: contains datablocks I. DOI: 10.1107/S160053680901054X/at2746Isup2.hkl

Additional supplementary materials:  crystallographic information; 3D view; checkCIF report

## Figures and Tables

**Table 1:** Hydrogen-bond geometry (Å, °)

*D*—H⋯*A*	*D*—H	H⋯*A*	*D*⋯*A*	*D*—H⋯*A*
O1*A*—H1*OA*⋯O2*B*^i^	0.91 (2)	1.80 (2)	2.711 (1)	179 (3)
O1*B*—H1*OB*⋯O2*A*^ii^	0.91 (2)	1.81 (2)	2.716 (1)	176 (2)
C4*A*—H4*AA*⋯O2*B*^i^	0.93	2.50	3.185 (1)	131
C4*B*—H4*BA*⋯O2*A*^ii^	0.93	2.50	3.192 (1)	131
C14*B*—H14*B*⋯O3*A*	0.93	2.56	3.485 (1)	173
C18*B*—H18*C*⋯*Cg*1^iii^	0.97	2.85	3.696 (1)	146
C24*B*—H24*D*⋯*Cg*2^iii^	0.97	2.71	3.554 (1)	145
C22*A*—H22*B*⋯*Cg*3^iv^	0.97	2.95	3.743 (1)	140

Symmetry codes: (i) 
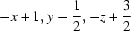
; (ii) 
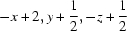
; (iii) 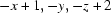; (iv) 
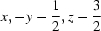
. *Cg*1, *Cg*2 and *Cg*3 are the centroids of the C1*A*–C6*A*, C10*B*–C15*B* and C1*B*–C6*B* rings, respectively.

**Fig. 1.**
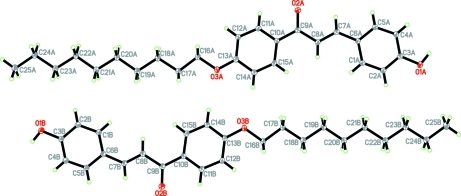
The asymmetric unit of the title compound, showing 50% probability displacement ellipsoids and the atom numbering scheme.

**Fig. 2.**
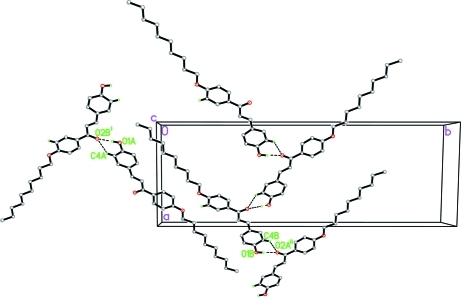
Part of the crystal packing of (I) showing R12(6) ring motifs generated by intermolecular interactions. The symmetry codes are given in Table 2.

## supplementary crystallographic information

### Comment

Chalcone derivatives are reported to possess biological properties such as anticancer (Bhat *et al.*, 2005), antimalarial (Xue *et al.*, 2004), antioxidant and antimicrobial activities (Yayli *et al.*, 2006), antiplatelet activity (Zhao *et al.*, 2005) as well as antihyperglycemic activity (Satyanarayana *et al.*, 2004). Chalcone derivatives possessing alkyl chains have been synthesized in our lab and their antibacterial activities were tested against *E. coli* ATCC 8739. All the synthesized chalcone derivatives showed antimicrobial activity. The structure reported in this paper, (I), is one of the chalcone derivatives mentioned above.

There are two crystallographically independent molecules (A and B) in the asymmetric unit (Fig. 1). The bond lengths observed in (I) show normal values as reported by Allen *et al.*, 1987. These two molecules (A and B) are interconnected by C14B—H14B···O3A intermolecular interactions (Table 1). In molecule A, the mean plane through the enone moiety (O2C7C8C9) and the two benzene rings make dihedral angles of 0.59 (7)° (C1—C6) and 4.49 (6)° (C10—C15) whereas in B, these angles are 4.21 (7)° (C1—C6) and 8.66 (7)° (C10—C15). The dihedral angles between the two benzene rings are 5.08 (5)° for molecule A and 9.23 (5)° for B. The alkoxyl tail in both molecules is coplanar with the attached ring with the torsion angle (C16—O3—C13—C12) in molecule B [5.06 (15)°] larger than in A [1.79 (15)°].

The enone moieties of both molecules adopt *s*-*cis* configuration with C7—C8—C9—O2 torsion angle being -1.2 (2)° for molecule A and -7.3 (2)° for B. The widening of C1A—C6A—C7A (123.94 (9)°) and C6A—C7A—C8A (128.28 (9)°) angles in molecule A is the result of the short H1AA···H8AA(2.32 Å) contact whereas short H8AA···H15A (2.17 Å) contact widened the C9A—C10A—C15A (124.53 (9)°). Similarly in molecule B, close interatomic contact between H1BA and H8BA (2.35 Å) results in the widening of C1B—C6B—C7B (124.33 (9)°) and C6B—C7B—C8B (129.01 (10)°) angles whereas the opening of C9B—C10B—C15B angle to 124.13 (9)° is the result of the close H8BA···H15B (2.18 Å) contact. Similar feature was also discussed in structures reported by Ng, Razak *et al.* (2006) and Ng, Patil *et al.* (2006).

In the crystal structure, O1A—H1OA···O2B^i^ and C4A—H4AA···O2B^i^ interactions in molecule A and O1B—H1OB···O2A^ii^ and C4B—H4BA···O2A^ii^ in B (Table 1) form bifurcated acceptor bonds which generate *R*^1^_2_(6) ring motifs (Fig. 2). These intermolecular interactions translate the molecules into one-dimensional extended chains along the [1 0 -1] direction. The crystal structure is further stabilized by C—H···π interactions (Table 1).

### Experimental

A mixture of 4-hydroxybenzaldehyde (2.44 g, 20 mmol) and 4-decyloxyacethophenone (5.53 ml, 20 mmol) and KOH (4.04 g, 72 mmol) in 60 ml of methanol was heated at reflux for 24 h. The reaction was cooled to room temperature and was acidified with cold diluted HCl (2 N). The resulting precipitate was filtered, washed and dried. The precipitate was dissolved in hexane–ethanol (7:1) mixture. After a few days of slow evaporation, crystals suitable for X-ray analysis were collected.

### Refinement

All the carbon-bound H atoms were positioned geometrically and refined using a riding model with C—H = 0.93–0.97 Å. The *U*_iso_ values were constrained to be U_iso_(H) =1.5U_equ_ (methyl H atoms) and U_iso_(H) =1.2U_equ_ (other H atoms). The rotating model group was considered for the methyl group. In the case of O1A and O1B, the hydrogen atoms were located from a difference Fourier map and refined isotropically.

### Crystal data

**Table:**

C_25_H_32_O_3_	*F*(000) = 1648
*M**_r_* = 380.51	*D*_x_ = 1.182 Mg m^−^^3^
Monoclinic, *P*2_1_/*c*	Mo *K*α radiation, λ = 0.71073 Å
Hall symbol: -P 2ybc	Cell parameters from 9788 reflections
*a* = 12.4437 (2) Å	θ = 2.4–33.6°
*b* = 35.5191 (6) Å	µ = 0.08 mm^−^^1^
*c* = 9.8004 (2) Å	*T* = 100 K
β = 99.284 (1)°	Block, colourless
*V* = 4274.93 (13) Å^3^	0.52 × 0.44 × 0.35 mm
*Z* = 8	

### Data collection

**Table:**

Bruker APEXII CCD area-detector diffractometer	16928 independent reflections
Radiation source: sealed tube	12634 reflections with *I* > 2σ(*I*)
graphite	*R*_int_ = 0.027
φ and ω scans	θ_max_ = 33.6°, θ_min_ = 1.2°
Absorption correction: multi-scan (*SADABS*; Bruker, 2005)	*h* = −19→12
*T*_min_ = 0.962, *T*_max_ = 0.974	*k* = −47→55
62626 measured reflections	*l* = −15→15

### Refinement

**Table:**

Refinement on *F*^2^	Primary atom site location: structure-invariant direct methods
Least-squares matrix: full	Secondary atom site location: difference Fourier map
*R*[*F*^2^ > 2σ(*F*^2^)] = 0.056	Hydrogen site location: inferred from neighbouring sites
*wR*(*F*^2^) = 0.153	H atoms treated by a mixture of independent and constrained refinement
*S* = 1.04	*w* = 1/[σ^2^(*F*_o_^2^) + (0.0694*P*)^2^ + 1.5875*P*] where *P* = (*F*_o_^2^ + 2*F*_c_^2^)/3
16928 reflections	(Δ/σ)_max_ = 0.001
515 parameters	Δρ_max_ = 0.54 e Å^−^^3^
0 restraints	Δρ_min_ = −0.25 e Å^−^^3^

### Special details

**Table:**

Experimental. The crystal was placed in the cold stream of an Oxford Cryosystems Cobra open-flow nitrogen cryostat (Cosier & Glazer, 1986) operating at 100.0 (1) K.
Geometry. All e.s.d.'s (except the e.s.d. in the dihedral angle between two l.s. planes) are estimated using the full covariance matrix. The cell e.s.d.'s are taken into account individually in the estimation of e.s.d.'s in distances, angles and torsion angles; correlations between e.s.d.'s in cell parameters are only used when they are defined by crystal symmetry. An approximate (isotropic) treatment of cell e.s.d.'s is used for estimating e.s.d.'s involving l.s. planes.
Refinement. Refinement of *F*^2^ against ALL reflections. The weighted *R*-factor *wR* and goodness of fit *S* are based on *F*^2^, conventional *R*-factors *R* are based on *F*, with *F* set to zero for negative *F*^2^. The threshold expression of *F*^2^ > σ(*F*^2^) is used only for calculating *R*-factors(gt) *etc*. and is not relevant to the choice of reflections for refinement. *R*-factors based on *F*^2^ are statistically about twice as large as those based on *F*, and *R*- factors based on ALL data will be even larger.

### Fractional atomic coordinates and isotropic or equivalent isotropic displacement parameters (Å^2^)

**Table:**

	*x*	*y*	*z*	*U*_iso_*/*U*_eq_	
O1A	0.19648 (7)	−0.12225 (2)	0.96568 (9)	0.02036 (16)	
O2A	0.68305 (7)	−0.08504 (2)	0.48100 (9)	0.02114 (16)	
O3A	0.86470 (6)	0.07913 (2)	0.43003 (8)	0.01776 (15)	
C1A	0.39100 (9)	−0.06947 (3)	0.80269 (10)	0.01565 (18)	
H1AA	0.4143	−0.0447	0.7989	0.019*	
C2A	0.31110 (9)	−0.07833 (3)	0.88043 (11)	0.01666 (18)	
H2AA	0.2810	−0.0595	0.9282	0.020*	
C3A	0.27532 (8)	−0.11552 (3)	0.88759 (10)	0.01508 (17)	
C4A	0.32006 (9)	−0.14375 (3)	0.81526 (11)	0.01803 (19)	
H4AA	0.2966	−0.1685	0.8192	0.022*	
C5A	0.39998 (9)	−0.13443 (3)	0.73730 (11)	0.01765 (19)	
H5AA	0.4295	−0.1533	0.6890	0.021*	
C6A	0.43747 (8)	−0.09747 (3)	0.72928 (10)	0.01372 (17)	
C7A	0.52133 (8)	−0.09029 (3)	0.64537 (10)	0.01487 (17)	
H7AA	0.5451	−0.1111	0.6011	0.018*	
C8A	0.56916 (8)	−0.05727 (3)	0.62344 (10)	0.01469 (17)	
H8AA	0.5488	−0.0353	0.6644	0.018*	
C9A	0.65358 (8)	−0.05601 (3)	0.53460 (10)	0.01421 (17)	
C10A	0.70600 (8)	−0.01986 (3)	0.50805 (10)	0.01370 (17)	
C11A	0.79143 (9)	−0.02091 (3)	0.43098 (11)	0.01715 (19)	
H11A	0.8116	−0.0439	0.3977	0.021*	
C12A	0.84675 (9)	0.01122 (3)	0.40270 (11)	0.01749 (19)	
H12A	0.9038	0.0097	0.3522	0.021*	
C13A	0.81582 (8)	0.04600 (3)	0.45113 (10)	0.01456 (17)	
C14A	0.72985 (8)	0.04794 (3)	0.52714 (11)	0.01556 (18)	
H14A	0.7089	0.0711	0.5588	0.019*	
C15A	0.67605 (8)	0.01542 (3)	0.55516 (10)	0.01479 (17)	
H15A	0.6192	0.0169	0.6059	0.018*	
C16A	0.95178 (9)	0.07828 (3)	0.34939 (11)	0.01798 (19)	
H16A	0.9254	0.0681	0.2583	0.022*	
H16B	1.0102	0.0623	0.3940	0.022*	
C17A	0.99332 (9)	0.11775 (3)	0.33678 (11)	0.01747 (19)	
H17A	1.0190	0.1279	0.4281	0.021*	
H17B	0.9347	0.1337	0.2921	0.021*	
C18A	1.08613 (9)	0.11765 (3)	0.25216 (12)	0.0205 (2)	
H18A	1.1456	0.1027	0.3004	0.025*	
H18B	1.0610	0.1056	0.1640	0.025*	
C19A	1.12904 (9)	0.15667 (3)	0.22545 (11)	0.01802 (19)	
H19A	1.1530	0.1691	0.3132	0.022*	
H19B	1.0705	0.1715	0.1744	0.022*	
C20A	1.22377 (9)	0.15512 (3)	0.14381 (12)	0.0193 (2)	
H20A	1.2836	0.1415	0.1975	0.023*	
H20B	1.2009	0.1412	0.0588	0.023*	
C21A	1.26404 (9)	0.19380 (3)	0.10818 (11)	0.01678 (18)	
H21A	1.2887	0.2074	0.1932	0.020*	
H21B	1.2037	0.2077	0.0565	0.020*	
C22A	1.35682 (9)	0.19220 (3)	0.02342 (11)	0.01782 (19)	
H22A	1.4177	0.1788	0.0759	0.021*	
H22B	1.3327	0.1781	−0.0607	0.021*	
C23A	1.39557 (9)	0.23103 (3)	−0.01474 (11)	0.01709 (18)	
H23A	1.3347	0.2443	−0.0680	0.021*	
H23B	1.4185	0.2452	0.0695	0.021*	
C24A	1.48909 (10)	0.23004 (3)	−0.09762 (13)	0.0224 (2)	
H24A	1.4664	0.2162	−0.1827	0.027*	
H24B	1.5503	0.2168	−0.0449	0.027*	
C25A	1.52534 (10)	0.26954 (3)	−0.13242 (13)	0.0231 (2)	
H25A	1.5845	0.2677	−0.1840	0.035*	
H25B	1.5488	0.2832	−0.0484	0.035*	
H25C	1.4655	0.2825	−0.1867	0.035*	
O1B	1.31221 (7)	0.33893 (2)	0.04708 (9)	0.02207 (17)	
O2B	0.84313 (8)	0.30275 (2)	0.55634 (10)	0.0293 (2)	
O3B	0.68508 (7)	0.13810 (2)	0.65982 (8)	0.01930 (16)	
C1B	1.12915 (9)	0.28531 (3)	0.22651 (12)	0.0189 (2)	
H1BA	1.1138	0.2602	0.2417	0.023*	
C2B	1.20746 (10)	0.29428 (3)	0.14624 (12)	0.0208 (2)	
H2BA	1.2436	0.2752	0.1071	0.025*	
C3B	1.23247 (9)	0.33191 (3)	0.12373 (11)	0.01589 (18)	
C4B	1.17622 (9)	0.36043 (3)	0.17965 (11)	0.01771 (19)	
H4BA	1.1914	0.3856	0.1640	0.021*	
C5B	1.09736 (9)	0.35102 (3)	0.25890 (11)	0.01814 (19)	
H5BA	1.0598	0.3702	0.2956	0.022*	
C6B	1.07262 (8)	0.31357 (3)	0.28527 (11)	0.01511 (18)	
C7B	0.99147 (9)	0.30651 (3)	0.37343 (11)	0.01700 (19)	
H7BA	0.9566	0.3278	0.4005	0.020*	
C8B	0.95974 (9)	0.27355 (3)	0.42135 (11)	0.01687 (18)	
H8BA	0.9904	0.2511	0.3971	0.020*	
C9B	0.87663 (9)	0.27302 (3)	0.51200 (11)	0.01733 (19)	
C10B	0.83094 (8)	0.23694 (3)	0.55219 (11)	0.01535 (18)	
C11B	0.75851 (9)	0.23806 (3)	0.64765 (11)	0.01700 (19)	
H11B	0.7433	0.2611	0.6854	0.020*	
C12B	0.70886 (9)	0.20588 (3)	0.68738 (11)	0.01700 (19)	
H12B	0.6610	0.2073	0.7509	0.020*	
C13B	0.73143 (8)	0.17123 (3)	0.63101 (10)	0.01518 (17)	
C14B	0.80510 (9)	0.16922 (3)	0.53728 (12)	0.0188 (2)	
H14B	0.8212	0.1461	0.5011	0.023*	
C15B	0.85396 (9)	0.20172 (3)	0.49851 (11)	0.01832 (19)	
H15B	0.9027	0.2002	0.4361	0.022*	
C16B	0.60126 (9)	0.13979 (3)	0.74562 (11)	0.01719 (19)	
H16C	0.5419	0.1556	0.7026	0.021*	
H16D	0.6305	0.1504	0.8351	0.021*	
C17B	0.56056 (9)	0.10029 (3)	0.76309 (11)	0.01687 (18)	
H17C	0.6200	0.0848	0.8081	0.020*	
H17D	0.5342	0.0895	0.6730	0.020*	
C18B	0.46876 (9)	0.10052 (3)	0.84962 (11)	0.01672 (18)	
H18C	0.4952	0.1120	0.9384	0.020*	
H18D	0.4094	0.1159	0.8033	0.020*	
C19B	0.42533 (9)	0.06142 (3)	0.87367 (11)	0.01718 (19)	
H19C	0.4847	0.0459	0.9189	0.021*	
H19D	0.3977	0.0500	0.7850	0.021*	
C20B	0.33482 (9)	0.06196 (3)	0.96191 (11)	0.01738 (19)	
H20C	0.3617	0.0742	1.0492	0.021*	
H20D	0.2745	0.0768	0.9151	0.021*	
C21B	0.29339 (9)	0.02284 (3)	0.99067 (11)	0.01762 (19)	
H21C	0.3540	0.0080	1.0364	0.021*	
H21D	0.2660	0.0108	0.9032	0.021*	
C22B	0.20355 (8)	0.02275 (3)	1.08003 (11)	0.01643 (18)	
H22C	0.2313	0.0342	1.1686	0.020*	
H22D	0.1434	0.0381	1.0356	0.020*	
C23B	0.16144 (8)	−0.01652 (3)	1.10443 (11)	0.01573 (18)	
H23C	0.2216	−0.0317	1.1498	0.019*	
H23D	0.1349	−0.0280	1.0156	0.019*	
C24B	0.07056 (9)	−0.01719 (3)	1.19178 (11)	0.01754 (19)	
H24C	0.0101	−0.0020	1.1471	0.021*	
H24D	0.0969	−0.0061	1.2814	0.021*	
C25B	0.03045 (10)	−0.05710 (3)	1.21225 (13)	0.0231 (2)	
H25D	−0.0276	−0.0562	1.2659	0.035*	
H25E	0.0893	−0.0719	1.2599	0.035*	
H25F	0.0044	−0.0682	1.1238	0.035*	
H1OB	1.3164 (14)	0.3643 (6)	0.0362 (19)	0.044 (5)*	
H1OA	0.1825 (15)	−0.1475 (6)	0.9592 (19)	0.046 (5)*	

### Atomic displacement parameters (Å^2^)

**Table:**

	*U*^11^	*U*^22^	*U*^33^	*U*^12^	*U*^13^	*U*^23^
O1A	0.0220 (4)	0.0178 (4)	0.0250 (4)	−0.0022 (3)	0.0151 (3)	0.0001 (3)
O2A	0.0248 (4)	0.0131 (3)	0.0295 (4)	−0.0007 (3)	0.0166 (3)	−0.0028 (3)
O3A	0.0201 (4)	0.0131 (3)	0.0229 (4)	−0.0027 (3)	0.0118 (3)	0.0005 (3)
C1A	0.0186 (5)	0.0123 (4)	0.0176 (4)	−0.0009 (3)	0.0077 (3)	−0.0004 (3)
C2A	0.0197 (5)	0.0134 (4)	0.0190 (4)	−0.0003 (3)	0.0092 (4)	−0.0017 (3)
C3A	0.0146 (4)	0.0154 (4)	0.0167 (4)	−0.0003 (3)	0.0069 (3)	0.0011 (3)
C4A	0.0206 (5)	0.0126 (4)	0.0236 (5)	−0.0017 (4)	0.0117 (4)	−0.0001 (3)
C5A	0.0205 (5)	0.0129 (4)	0.0220 (5)	−0.0003 (3)	0.0110 (4)	−0.0012 (3)
C6A	0.0145 (4)	0.0126 (4)	0.0153 (4)	−0.0003 (3)	0.0060 (3)	0.0004 (3)
C7A	0.0152 (4)	0.0143 (4)	0.0164 (4)	0.0003 (3)	0.0065 (3)	0.0003 (3)
C8A	0.0154 (4)	0.0138 (4)	0.0163 (4)	−0.0005 (3)	0.0068 (3)	−0.0009 (3)
C9A	0.0143 (4)	0.0133 (4)	0.0161 (4)	−0.0003 (3)	0.0055 (3)	0.0004 (3)
C10A	0.0147 (4)	0.0124 (4)	0.0152 (4)	−0.0002 (3)	0.0059 (3)	0.0001 (3)
C11A	0.0191 (5)	0.0135 (4)	0.0212 (5)	−0.0007 (3)	0.0102 (4)	−0.0018 (3)
C12A	0.0187 (5)	0.0148 (4)	0.0217 (5)	−0.0015 (4)	0.0116 (4)	−0.0006 (3)
C13A	0.0154 (4)	0.0133 (4)	0.0159 (4)	−0.0012 (3)	0.0055 (3)	0.0012 (3)
C14A	0.0166 (5)	0.0132 (4)	0.0183 (4)	0.0004 (3)	0.0068 (3)	−0.0004 (3)
C15A	0.0144 (4)	0.0148 (4)	0.0168 (4)	0.0001 (3)	0.0073 (3)	0.0005 (3)
C16A	0.0175 (5)	0.0161 (5)	0.0226 (5)	−0.0013 (4)	0.0103 (4)	0.0016 (4)
C17A	0.0176 (5)	0.0155 (4)	0.0208 (5)	−0.0024 (3)	0.0075 (4)	0.0011 (3)
C18A	0.0191 (5)	0.0165 (5)	0.0284 (5)	−0.0011 (4)	0.0117 (4)	0.0028 (4)
C19A	0.0189 (5)	0.0161 (5)	0.0207 (5)	−0.0016 (4)	0.0082 (4)	0.0020 (3)
C20A	0.0199 (5)	0.0159 (5)	0.0242 (5)	−0.0016 (4)	0.0098 (4)	0.0026 (4)
C21A	0.0163 (5)	0.0162 (4)	0.0192 (4)	−0.0021 (3)	0.0070 (4)	0.0004 (3)
C22A	0.0181 (5)	0.0165 (5)	0.0208 (5)	−0.0016 (4)	0.0087 (4)	0.0004 (3)
C23A	0.0176 (5)	0.0163 (4)	0.0192 (4)	−0.0012 (3)	0.0087 (4)	0.0000 (3)
C24A	0.0220 (5)	0.0189 (5)	0.0300 (6)	0.0005 (4)	0.0152 (4)	0.0025 (4)
C25A	0.0214 (5)	0.0216 (5)	0.0282 (5)	−0.0036 (4)	0.0100 (4)	0.0036 (4)
O1B	0.0255 (4)	0.0154 (4)	0.0301 (4)	−0.0002 (3)	0.0187 (3)	0.0018 (3)
O2B	0.0372 (5)	0.0137 (4)	0.0445 (5)	−0.0007 (3)	0.0292 (4)	−0.0017 (3)
O3B	0.0230 (4)	0.0129 (3)	0.0256 (4)	−0.0035 (3)	0.0146 (3)	−0.0003 (3)
C1B	0.0219 (5)	0.0120 (4)	0.0257 (5)	−0.0013 (4)	0.0129 (4)	−0.0010 (4)
C2B	0.0260 (6)	0.0131 (4)	0.0271 (5)	0.0005 (4)	0.0161 (4)	−0.0015 (4)
C3B	0.0169 (5)	0.0143 (4)	0.0185 (4)	−0.0004 (3)	0.0089 (3)	0.0005 (3)
C4B	0.0208 (5)	0.0118 (4)	0.0230 (5)	0.0006 (3)	0.0109 (4)	0.0015 (3)
C5B	0.0205 (5)	0.0123 (4)	0.0243 (5)	0.0012 (3)	0.0116 (4)	0.0011 (3)
C6B	0.0143 (4)	0.0137 (4)	0.0187 (4)	0.0002 (3)	0.0070 (3)	0.0008 (3)
C7B	0.0168 (5)	0.0149 (4)	0.0211 (5)	0.0001 (3)	0.0087 (4)	0.0001 (3)
C8B	0.0163 (5)	0.0143 (4)	0.0220 (5)	−0.0012 (3)	0.0090 (4)	−0.0010 (3)
C9B	0.0176 (5)	0.0147 (4)	0.0219 (5)	−0.0008 (3)	0.0099 (4)	−0.0003 (3)
C10B	0.0149 (4)	0.0137 (4)	0.0191 (4)	−0.0014 (3)	0.0076 (3)	−0.0001 (3)
C11B	0.0184 (5)	0.0137 (4)	0.0213 (5)	−0.0017 (3)	0.0103 (4)	−0.0019 (3)
C12B	0.0183 (5)	0.0154 (4)	0.0199 (4)	−0.0022 (3)	0.0108 (4)	−0.0008 (3)
C13B	0.0158 (4)	0.0131 (4)	0.0180 (4)	−0.0017 (3)	0.0066 (3)	0.0008 (3)
C14B	0.0214 (5)	0.0132 (4)	0.0248 (5)	−0.0012 (4)	0.0122 (4)	−0.0022 (4)
C15B	0.0199 (5)	0.0148 (4)	0.0233 (5)	−0.0014 (4)	0.0126 (4)	−0.0009 (4)
C16B	0.0186 (5)	0.0155 (4)	0.0198 (4)	−0.0022 (4)	0.0101 (4)	0.0006 (3)
C17B	0.0191 (5)	0.0141 (4)	0.0193 (4)	−0.0024 (3)	0.0087 (4)	0.0013 (3)
C18B	0.0179 (5)	0.0144 (4)	0.0190 (4)	−0.0015 (3)	0.0065 (4)	0.0015 (3)
C19B	0.0195 (5)	0.0152 (4)	0.0183 (4)	−0.0030 (4)	0.0075 (4)	−0.0003 (3)
C20B	0.0191 (5)	0.0147 (4)	0.0199 (4)	−0.0017 (4)	0.0080 (4)	0.0011 (3)
C21B	0.0200 (5)	0.0148 (4)	0.0199 (4)	−0.0023 (4)	0.0086 (4)	0.0008 (3)
C22B	0.0164 (5)	0.0157 (4)	0.0185 (4)	−0.0017 (3)	0.0067 (3)	0.0008 (3)
C23B	0.0166 (4)	0.0143 (4)	0.0178 (4)	−0.0006 (3)	0.0070 (3)	0.0004 (3)
C24B	0.0174 (5)	0.0171 (5)	0.0199 (5)	−0.0013 (4)	0.0083 (4)	0.0011 (3)
C25B	0.0227 (5)	0.0211 (5)	0.0273 (5)	−0.0056 (4)	0.0094 (4)	0.0019 (4)

### Geometric parameters (Å, °)

**Table:**

O1A—C3A	1.3592 (12)	O1B—C3B	1.3607 (12)
O1A—H1OA	0.92 (2)	O1B—H1OB	0.91 (2)
O2A—C9A	1.2396 (12)	O2B—C9B	1.2396 (13)
O3A—C13A	1.3559 (12)	O3B—C13B	1.3600 (12)
O3A—C16A	1.4407 (12)	O3B—C16B	1.4427 (12)
C1A—C2A	1.3829 (14)	C1B—C2B	1.3848 (14)
C1A—C6A	1.4054 (14)	C1B—C6B	1.4016 (14)
C1A—H1AA	0.9300	C1B—H1BA	0.9300
C2A—C3A	1.3995 (14)	C2B—C3B	1.3980 (14)
C2A—H2AA	0.9300	C2B—H2BA	0.9300
C3A—C4A	1.3946 (14)	C3B—C4B	1.3928 (14)
C4A—C5A	1.3884 (14)	C4B—C5B	1.3871 (14)
C4A—H4AA	0.9300	C4B—H4BA	0.9300
C5A—C6A	1.3998 (14)	C5B—C6B	1.3989 (14)
C5A—H5AA	0.9300	C5B—H5BA	0.9300
C6A—C7A	1.4516 (13)	C6B—C7B	1.4527 (14)
C7A—C8A	1.3480 (14)	C7B—C8B	1.3445 (14)
C7A—H7AA	0.9300	C7B—H7BA	0.9300
C8A—C9A	1.4693 (13)	C8B—C9B	1.4681 (14)
C8A—H8AA	0.9300	C8B—H8BA	0.9300
C9A—C10A	1.4821 (14)	C9B—C10B	1.4810 (14)
C10A—C11A	1.4004 (13)	C10B—C11B	1.4005 (14)
C10A—C15A	1.4062 (14)	C10B—C15B	1.4044 (14)
C11A—C12A	1.3837 (14)	C11B—C12B	1.3848 (14)
C11A—H11A	0.9300	C11B—H11B	0.9300
C12A—C13A	1.3994 (14)	C12B—C13B	1.3958 (14)
C12A—H12A	0.9300	C12B—H12B	0.9300
C13A—C14A	1.4005 (14)	C13B—C14B	1.4004 (14)
C14A—C15A	1.3846 (14)	C14B—C15B	1.3860 (15)
C14A—H14A	0.9300	C14B—H14B	0.9300
C15A—H15A	0.9300	C15B—H15B	0.9300
C16A—C17A	1.5060 (14)	C16B—C17B	1.5106 (14)
C16A—H16A	0.9700	C16B—H16C	0.9700
C16A—H16B	0.9700	C16B—H16D	0.9700
C17A—C18A	1.5266 (15)	C17B—C18B	1.5286 (14)
C17A—H17A	0.9700	C17B—H17C	0.9700
C17A—H17B	0.9700	C17B—H17D	0.9700
C18A—C19A	1.5231 (15)	C18B—C19B	1.5224 (14)
C18A—H18A	0.9700	C18B—H18C	0.9700
C18A—H18B	0.9700	C18B—H18D	0.9700
C19A—C20A	1.5287 (15)	C19B—C20B	1.5267 (14)
C19A—H19A	0.9700	C19B—H19C	0.9700
C19A—H19B	0.9700	C19B—H19D	0.9700
C20A—C21A	1.5223 (14)	C20B—C21B	1.5239 (14)
C20A—H20A	0.9700	C20B—H20C	0.9700
C20A—H20B	0.9700	C20B—H20D	0.9700
C21A—C22A	1.5282 (14)	C21B—C22B	1.5272 (14)
C21A—H21A	0.9700	C21B—H21C	0.9700
C21A—H21B	0.9700	C21B—H21D	0.9700
C22A—C23A	1.5272 (15)	C22B—C23B	1.5223 (14)
C22A—H22A	0.9700	C22B—H22C	0.9700
C22A—H22B	0.9700	C22B—H22D	0.9700
C23A—C24A	1.5232 (14)	C23B—C24B	1.5247 (14)
C23A—H23A	0.9700	C23B—H23C	0.9700
C23A—H23B	0.9700	C23B—H23D	0.9700
C24A—C25A	1.5291 (16)	C24B—C25B	1.5269 (15)
C24A—H24A	0.9700	C24B—H24C	0.9700
C24A—H24B	0.9700	C24B—H24D	0.9700
C25A—H25A	0.9600	C25B—H25D	0.9600
C25A—H25B	0.9600	C25B—H25E	0.9600
C25A—H25C	0.9600	C25B—H25F	0.9600
			
C3A—O1A—H1OA	106.3 (11)	C3B—O1B—H1OB	107.9 (11)
C13A—O3A—C16A	117.46 (8)	C13B—O3B—C16B	117.12 (8)
C2A—C1A—C6A	120.85 (9)	C2B—C1B—C6B	120.96 (10)
C2A—C1A—H1AA	119.6	C2B—C1B—H1BA	119.5
C6A—C1A—H1AA	119.6	C6B—C1B—H1BA	119.5
C1A—C2A—C3A	120.35 (9)	C1B—C2B—C3B	120.31 (10)
C1A—C2A—H2AA	119.8	C1B—C2B—H2BA	119.8
C3A—C2A—H2AA	119.8	C3B—C2B—H2BA	119.8
O1A—C3A—C4A	122.92 (9)	O1B—C3B—C4B	122.79 (9)
O1A—C3A—C2A	117.29 (9)	O1B—C3B—C2B	117.58 (9)
C4A—C3A—C2A	119.79 (9)	C4B—C3B—C2B	119.64 (9)
C5A—C4A—C3A	119.24 (9)	C5B—C4B—C3B	119.39 (9)
C5A—C4A—H4AA	120.4	C5B—C4B—H4BA	120.3
C3A—C4A—H4AA	120.4	C3B—C4B—H4BA	120.3
C4A—C5A—C6A	121.95 (9)	C4B—C5B—C6B	121.96 (9)
C4A—C5A—H5AA	119.0	C4B—C5B—H5BA	119.0
C6A—C5A—H5AA	119.0	C6B—C5B—H5BA	119.0
C5A—C6A—C1A	117.82 (9)	C5B—C6B—C1B	117.72 (9)
C5A—C6A—C7A	118.24 (9)	C5B—C6B—C7B	117.95 (9)
C1A—C6A—C7A	123.94 (9)	C1B—C6B—C7B	124.33 (9)
C8A—C7A—C6A	128.28 (9)	C8B—C7B—C6B	129.01 (10)
C8A—C7A—H7AA	115.9	C8B—C7B—H7BA	115.5
C6A—C7A—H7AA	115.9	C6B—C7B—H7BA	115.5
C7A—C8A—C9A	119.82 (9)	C7B—C8B—C9B	119.85 (9)
C7A—C8A—H8AA	120.1	C7B—C8B—H8BA	120.1
C9A—C8A—H8AA	120.1	C9B—C8B—H8BA	120.1
O2A—C9A—C8A	120.96 (9)	O2B—C9B—C8B	120.72 (9)
O2A—C9A—C10A	118.54 (9)	O2B—C9B—C10B	118.61 (9)
C8A—C9A—C10A	120.49 (8)	C8B—C9B—C10B	120.67 (9)
C11A—C10A—C15A	117.73 (9)	C11B—C10B—C15B	117.89 (9)
C11A—C10A—C9A	117.74 (9)	C11B—C10B—C9B	117.97 (9)
C15A—C10A—C9A	124.53 (9)	C15B—C10B—C9B	124.13 (9)
C12A—C11A—C10A	122.10 (9)	C12B—C11B—C10B	121.92 (9)
C12A—C11A—H11A	118.9	C12B—C11B—H11B	119.0
C10A—C11A—H11A	118.9	C10B—C11B—H11B	119.0
C11A—C12A—C13A	119.16 (9)	C11B—C12B—C13B	119.28 (9)
C11A—C12A—H12A	120.4	C11B—C12B—H12B	120.4
C13A—C12A—H12A	120.4	C13B—C12B—H12B	120.4
O3A—C13A—C12A	124.04 (9)	O3B—C13B—C12B	124.16 (9)
O3A—C13A—C14A	116.00 (9)	O3B—C13B—C14B	115.87 (9)
C12A—C13A—C14A	119.96 (9)	C12B—C13B—C14B	119.97 (9)
C15A—C14A—C13A	119.97 (9)	C15B—C14B—C13B	119.99 (9)
C15A—C14A—H14A	120.0	C15B—C14B—H14B	120.0
C13A—C14A—H14A	120.0	C13B—C14B—H14B	120.0
C14A—C15A—C10A	121.07 (9)	C14B—C15B—C10B	120.94 (9)
C14A—C15A—H15A	119.5	C14B—C15B—H15B	119.5
C10A—C15A—H15A	119.5	C10B—C15B—H15B	119.5
O3A—C16A—C17A	108.91 (8)	O3B—C16B—C17B	108.34 (8)
O3A—C16A—H16A	109.9	O3B—C16B—H16C	110.0
C17A—C16A—H16A	109.9	C17B—C16B—H16C	110.0
O3A—C16A—H16B	109.9	O3B—C16B—H16D	110.0
C17A—C16A—H16B	109.9	C17B—C16B—H16D	110.0
H16A—C16A—H16B	108.3	H16C—C16B—H16D	108.4
C16A—C17A—C18A	110.01 (9)	C16B—C17B—C18B	110.62 (8)
C16A—C17A—H17A	109.7	C16B—C17B—H17C	109.5
C18A—C17A—H17A	109.7	C18B—C17B—H17C	109.5
C16A—C17A—H17B	109.7	C16B—C17B—H17D	109.5
C18A—C17A—H17B	109.7	C18B—C17B—H17D	109.5
H17A—C17A—H17B	108.2	H17C—C17B—H17D	108.1
C19A—C18A—C17A	114.09 (9)	C19B—C18B—C17B	113.38 (9)
C19A—C18A—H18A	108.7	C19B—C18B—H18C	108.9
C17A—C18A—H18A	108.7	C17B—C18B—H18C	108.9
C19A—C18A—H18B	108.7	C19B—C18B—H18D	108.9
C17A—C18A—H18B	108.7	C17B—C18B—H18D	108.9
H18A—C18A—H18B	107.6	H18C—C18B—H18D	107.7
C18A—C19A—C20A	112.24 (9)	C18B—C19B—C20B	112.89 (9)
C18A—C19A—H19A	109.2	C18B—C19B—H19C	109.0
C20A—C19A—H19A	109.2	C20B—C19B—H19C	109.0
C18A—C19A—H19B	109.2	C18B—C19B—H19D	109.0
C20A—C19A—H19B	109.2	C20B—C19B—H19D	109.0
H19A—C19A—H19B	107.9	H19C—C19B—H19D	107.8
C21A—C20A—C19A	113.43 (9)	C21B—C20B—C19B	113.32 (9)
C21A—C20A—H20A	108.9	C21B—C20B—H20C	108.9
C19A—C20A—H20A	108.9	C19B—C20B—H20C	108.9
C21A—C20A—H20B	108.9	C21B—C20B—H20D	108.9
C19A—C20A—H20B	108.9	C19B—C20B—H20D	108.9
H20A—C20A—H20B	107.7	H20C—C20B—H20D	107.7
C20A—C21A—C22A	113.37 (9)	C20B—C21B—C22B	114.14 (9)
C20A—C21A—H21A	108.9	C20B—C21B—H21C	108.7
C22A—C21A—H21A	108.9	C22B—C21B—H21C	108.7
C20A—C21A—H21B	108.9	C20B—C21B—H21D	108.7
C22A—C21A—H21B	108.9	C22B—C21B—H21D	108.7
H21A—C21A—H21B	107.7	H21C—C21B—H21D	107.6
C23A—C22A—C21A	113.31 (9)	C23B—C22B—C21B	113.23 (8)
C23A—C22A—H22A	108.9	C23B—C22B—H22C	108.9
C21A—C22A—H22A	108.9	C21B—C22B—H22C	108.9
C23A—C22A—H22B	108.9	C23B—C22B—H22D	108.9
C21A—C22A—H22B	108.9	C21B—C22B—H22D	108.9
H22A—C22A—H22B	107.7	H22C—C22B—H22D	107.7
C24A—C23A—C22A	114.10 (9)	C22B—C23B—C24B	114.07 (8)
C24A—C23A—H23A	108.7	C22B—C23B—H23C	108.7
C22A—C23A—H23A	108.7	C24B—C23B—H23C	108.7
C24A—C23A—H23B	108.7	C22B—C23B—H23D	108.7
C22A—C23A—H23B	108.7	C24B—C23B—H23D	108.7
H23A—C23A—H23B	107.6	H23C—C23B—H23D	107.6
C23A—C24A—C25A	112.09 (9)	C23B—C24B—C25B	112.09 (9)
C23A—C24A—H24A	109.2	C23B—C24B—H24C	109.2
C25A—C24A—H24A	109.2	C25B—C24B—H24C	109.2
C23A—C24A—H24B	109.2	C23B—C24B—H24D	109.2
C25A—C24A—H24B	109.2	C25B—C24B—H24D	109.2
H24A—C24A—H24B	107.9	H24C—C24B—H24D	107.9
C24A—C25A—H25A	109.5	C24B—C25B—H25D	109.5
C24A—C25A—H25B	109.5	C24B—C25B—H25E	109.5
H25A—C25A—H25B	109.5	H25D—C25B—H25E	109.5
C24A—C25A—H25C	109.5	C24B—C25B—H25F	109.5
H25A—C25A—H25C	109.5	H25D—C25B—H25F	109.5
H25B—C25A—H25C	109.5	H25E—C25B—H25F	109.5
			
C6A—C1A—C2A—C3A	0.19 (16)	C6B—C1B—C2B—C3B	0.77 (18)
C1A—C2A—C3A—O1A	−179.91 (10)	C1B—C2B—C3B—O1B	178.25 (11)
C1A—C2A—C3A—C4A	−0.38 (16)	C1B—C2B—C3B—C4B	−1.61 (17)
O1A—C3A—C4A—C5A	179.69 (10)	O1B—C3B—C4B—C5B	−178.78 (10)
C2A—C3A—C4A—C5A	0.18 (16)	C2B—C3B—C4B—C5B	1.07 (17)
C3A—C4A—C5A—C6A	0.20 (17)	C3B—C4B—C5B—C6B	0.31 (17)
C4A—C5A—C6A—C1A	−0.38 (16)	C4B—C5B—C6B—C1B	−1.13 (17)
C4A—C5A—C6A—C7A	179.95 (10)	C4B—C5B—C6B—C7B	177.88 (10)
C2A—C1A—C6A—C5A	0.18 (15)	C2B—C1B—C6B—C5B	0.58 (17)
C2A—C1A—C6A—C7A	179.83 (10)	C2B—C1B—C6B—C7B	−178.35 (11)
C5A—C6A—C7A—C8A	−179.76 (11)	C5B—C6B—C7B—C8B	−174.80 (11)
C1A—C6A—C7A—C8A	0.58 (17)	C1B—C6B—C7B—C8B	4.13 (19)
C6A—C7A—C8A—C9A	179.70 (10)	C6B—C7B—C8B—C9B	178.96 (10)
C7A—C8A—C9A—O2A	−1.22 (16)	C7B—C8B—C9B—O2B	−7.31 (17)
C7A—C8A—C9A—C10A	179.25 (9)	C7B—C8B—C9B—C10B	172.58 (10)
O2A—C9A—C10A—C11A	−4.18 (15)	O2B—C9B—C10B—C11B	−5.15 (16)
C8A—C9A—C10A—C11A	175.37 (9)	C8B—C9B—C10B—C11B	174.95 (10)
O2A—C9A—C10A—C15A	175.97 (10)	O2B—C9B—C10B—C15B	173.51 (11)
C8A—C9A—C10A—C15A	−4.49 (15)	C8B—C9B—C10B—C15B	−6.39 (17)
C15A—C10A—C11A—C12A	0.99 (16)	C15B—C10B—C11B—C12B	−1.09 (16)
C9A—C10A—C11A—C12A	−178.88 (10)	C9B—C10B—C11B—C12B	177.65 (10)
C10A—C11A—C12A—C13A	−0.74 (17)	C10B—C11B—C12B—C13B	0.02 (17)
C16A—O3A—C13A—C12A	1.79 (15)	C16B—O3B—C13B—C12B	5.06 (15)
C16A—O3A—C13A—C14A	−178.55 (9)	C16B—O3B—C13B—C14B	−174.26 (9)
C11A—C12A—C13A—O3A	179.64 (10)	C11B—C12B—C13B—O3B	−178.14 (10)
C11A—C12A—C13A—C14A	0.00 (16)	C11B—C12B—C13B—C14B	1.16 (16)
O3A—C13A—C14A—C15A	−179.21 (9)	O3B—C13B—C14B—C15B	178.11 (10)
C12A—C13A—C14A—C15A	0.47 (16)	C12B—C13B—C14B—C15B	−1.24 (17)
C13A—C14A—C15A—C10A	−0.21 (16)	C13B—C14B—C15B—C10B	0.14 (17)
C11A—C10A—C15A—C14A	−0.50 (15)	C11B—C10B—C15B—C14B	1.00 (16)
C9A—C10A—C15A—C14A	179.35 (10)	C9B—C10B—C15B—C14B	−177.66 (11)
C13A—O3A—C16A—C17A	179.19 (9)	C13B—O3B—C16B—C17B	179.40 (9)
O3A—C16A—C17A—C18A	179.77 (9)	O3B—C16B—C17B—C18B	−178.19 (8)
C16A—C17A—C18A—C19A	176.24 (10)	C16B—C17B—C18B—C19B	−178.88 (9)
C17A—C18A—C19A—C20A	178.47 (9)	C17B—C18B—C19B—C20B	179.18 (9)
C18A—C19A—C20A—C21A	176.60 (9)	C18B—C19B—C20B—C21B	−178.06 (9)
C19A—C20A—C21A—C22A	−178.54 (9)	C19B—C20B—C21B—C22B	179.42 (9)
C20A—C21A—C22A—C23A	178.83 (9)	C20B—C21B—C22B—C23B	178.56 (9)
C21A—C22A—C23A—C24A	179.22 (9)	C21B—C22B—C23B—C24B	−179.19 (9)
C22A—C23A—C24A—C25A	−179.64 (10)	C22B—C23B—C24B—C25B	179.50 (9)

### Hydrogen-bond geometry (Å, °)

**Table:**

*D*—H···*A*	*D*—H	H···*A*	*D*···*A*	*D*—H···*A*
O1A—H1OA···O2B^i^	0.91 (2)	1.80 (2)	2.711 (1)	179 (3)
O1B—H1OB···O2A^ii^	0.91 (2)	1.81 (2)	2.716 (1)	176 (2)
C4A—H4AA···O2B^i^	0.93	2.50	3.185 (1)	131
C4B—H4BA···O2A^ii^	0.93	2.50	3.192 (1)	131
C14B—H14B···O3A	0.93	2.56	3.485 (1)	173
C18B—H18C···Cg1^iii^	0.97	2.85	3.696 (1)	146
C24B—H24D···Cg2^iii^	0.97	2.71	3.554 (1)	145
C22A—H22B···Cg3^iv^	0.97	2.95	3.743 (1)	140

Symmetry codes: (i) −*x*+1, *y*−1/2, −*z*+3/2; (ii) −*x*+2, *y*+1/2, −*z*+1/2; (iii) −*x*+1, −*y*, −*z*+2; (iv) *x*, −*y*−1/2, *z*−3/2.
